# Pathogenesis, Diagnosis, and Clinical Management of Aortic Aneurysms: Current Status and Future Perspectives

**DOI:** 10.31083/RCM49246

**Published:** 2026-06-26

**Authors:** Biyue Hu, Weilin Yang

**Affiliations:** ^1^Department of Radiology, West China School of Public Health and West China Fourth Hospital, Sichuan University, 610041 Chengdu, Sichuan, China; ^2^Department of Radiology, Key Laboratory of Birth Defects and Related Diseases of Women and Children (Sichuan University), Ministry of Education, West China Second University Hospital, Sichuan University, 610041 Chengdu, Sichuan, China

**Keywords:** aortic aneurysm, abdominal aortic aneurysm, thoracic aortic aneurysm, pathogenesis, diagnosis, management, endovascular repair, precision medicine

## Abstract

Aortic aneurysm (AA) is a life-threatening degenerative vascular disease defined as localized dilation of the aorta exceeding 1.5 times the normal diameter and isprimarily classified into abdominal aortic aneurysm (AAA) and thoracic aortic aneurysm (TAA). With an aging population and increased exposure to risk factors, the incidence of AA is rising, and rupture is associated with a mortality rate of 80–90%. Recent advances in molecular biology, imaging, and interventional techniques have expanded the understanding of AA pathogenesis beyond the traditional “degenerative disease” model to encompass genetic variation, extracellular matrix (ECM) remodeling, inflammatory responses, and dysregulated signaling pathway. Meanwhile, diagnostic approaches have become increasingly precise, enabling earlier detection through routine screening and three-dimensional vascular reconstruction. Clinical management has evolved into a comprehensive strategy centered on surgical intervention, supported by pharmacological therapy and long-term monitoring. This review systematically examines the pathophysiological mechanisms, advancements in diagnostic technologies, and evidence-based clinical management of AA, discusses current controversies, and explores future applications of precision medicine.

## 1. Introduction

The aorta, the body’s largest artery, is responsible for transporting oxygenated blood from the heart to the systemic circulation. Pathological dilation of this vessel can lead to life-threatening conditions, such as thoracic aortic aneurysms (TAAs), which are increasingly recognized as critical manifestations of cardiovascular aging [1,2]. In general, the development and progression of aortic aneurysms (AAs) involve multi-factorial mechanisms and require comprehensive management strategies [3]. These conditions range from hereditary thoracic aortic diseases to specific intraluminal processes driving abdominal aortic aneurysm (AAA) progression [4,5]. Ultimately, its structural integrity relies on the synergistic function of its three wall layers (the intima, media, and adventitia), and the essence of an AA is the progressive dilation resulting from the disruption of this vascular wall structure, driven by the combined effects of genetic predisposition, environmental risk factors, and dysfunctional vascular wall cells [6]. In the United States, AAA is a leading cause of death among the elderly, with significant annual mortality attributable to aneurysm rupture or dissection [2,7]. Although less common than AAA, TAA often presents with subtle early symptoms, and emergency treatment following rupture is more challenging, leading to poorer outcomes [8,9].

While traditionally viewed as an atherosclerotic degenerative disease, recent research confirms significant heterogeneity in AA pathogenesis: approximately 10% to 20% of cases are familial, involving mutations in genes such as *FBN1*, *COL3A1*, and *TGFBR1/2* [10]. Specifically, recent patient-specific stem cell models have provided profound insights into the lineage-specific smooth muscle cell defects driven by TGFBR1 mutations [11]. Sporadic cases are closely associated with environmental factors like smoking, hypertension, and metabolic syndrome [2,12]. Diagnostic techniques such as ultrasound, computed tomography angiography (CTA), and magnetic resonance angiography (MRA) enable early detection and assessment. Therapeutically, the adoption of endovascular aneurysm repair (EVAR) has significantly reduced operative mortality, establishing it as the preferred treatment for suitable AAA cases [13]. Similarly, thoracic endovascular aortic repair (TEVAR) has increasingly replaced open surgery for many TAA cases [1].

Despite these advancements, AA management faces several challenges, including the accurate identification of high-risk patients for rupture, effective pharmacological intervention to slow aneurysm progression, treatment strategy selection for complex anatomies, and long-term management of postoperative complications [3]. This article, based on recent significant research findings (2015–2025) and clinical guidelines, provides a systematic review from the core dimensions of pathological mechanisms, diagnostic techniques, and clinical management, discussing current controversies and future directions to offer new insights for basic research and clinical practice in AA.

## 2. Pathophysiology and Pathogenesis of Aortic Aneurysm

**Fig. 1.**
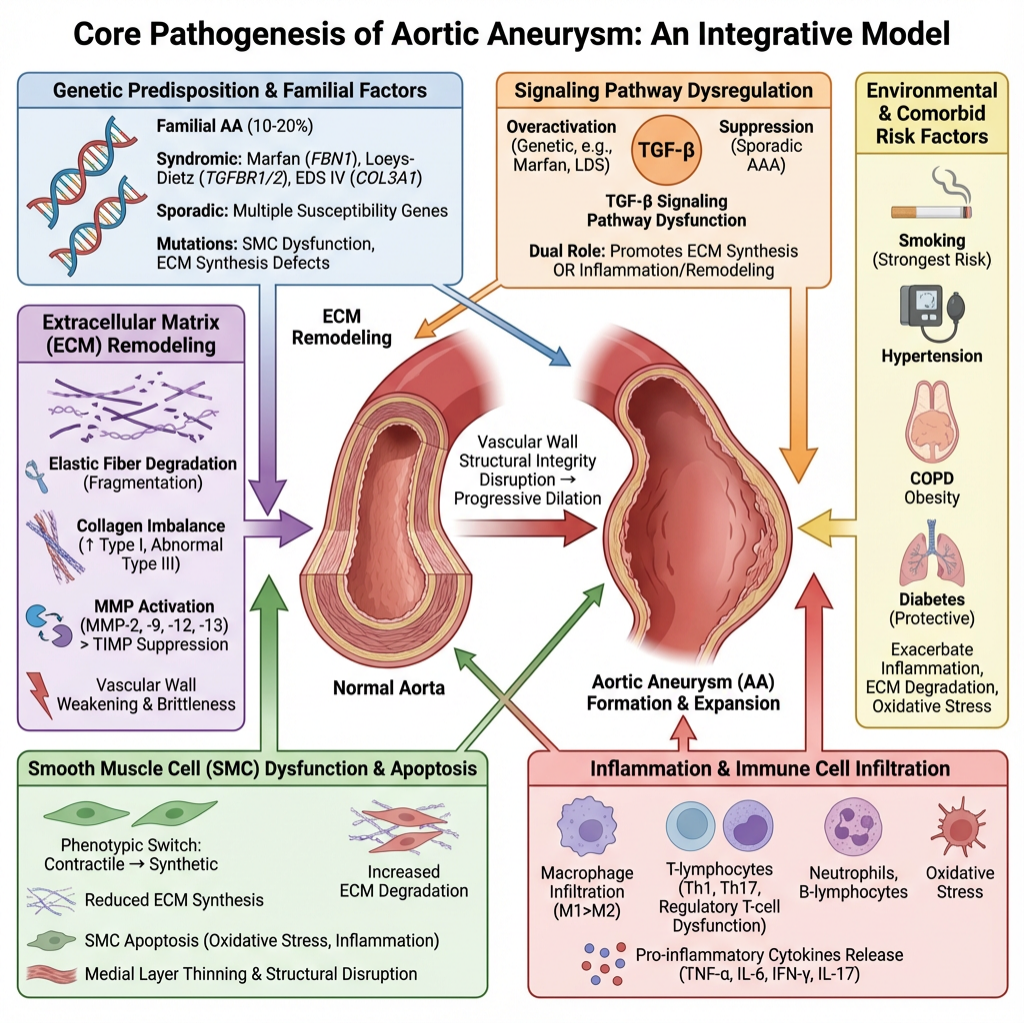
**Core pathogenic mechanisms of aortic aneurysm**. Aortic aneurysm formation is driven by the interplay of genetic predispositions, environmental risk factors, and dysfunctional vascular wall cells. Key processes include dysregulation of the TGF-β signaling pathway, inflammatory cell infiltration (e.g., macrophages, T cells) leading to protease release, degradation of the extracellular matrix (ECM) by matrix metalloproteinases (MMPs), and smooth muscle cell (SMC) apoptosis and phenotypic switching, collectively resulting in the loss of vascular wall integrity and progressive dilation. TGF-β, transforming growth factor-beta; LDS, Loeys-Dietz syndrome; AAA, abdominal aortic aneurysm; COPD, chronic obstructive pulmonary disease.

The pathogenesis of AA is a complex, multi-step process centered on the disruption of vascular wall structural integrity, involving intricate interactions between genetic factors, extracellular matrix (ECM) remodeling, inflammatory responses, and dysregulated signaling pathways (Fig. 1). Significant differences exist in the mechanistic characteristics of different subtypes (AAA and TAA) [6].

### 2.1 Genetic Factors and Familial Aortic Aneurysm

Genetic factors play a critical role in AA pathogenesis. The incidence of familial aortic aneurysm is approximately 3–4 times higher than that of sporadic cases. Well-defined genetic AA syndromes include Marfan syndrome (MFS), Loeys-Dietz syndrome (LDS), and Ehlers-Danlos syndrome type IV (EDS IV), caused by mutations in *FBN1*, *TGFBR1*/*TGFBR2*, and *COL3A1 *genes, respectively [11].

MFS, one of the most common inherited connective tissue disorders, results from *FBN1* mutations leading to defective fibrillin-1, impairing elastic fiber assembly and stability. Patients exhibit disrupted and disorganized elastic fibers in the aortic media, predisposing them to TAA and aortic dissection. LDS, a more severe autosomal dominant disorder caused by *TGFBR1* or *TGFBR2 *mutations, is characterized by arterial tortuosity and aneurysms, often accompanied by hypertelorism and bifid uvula. The risk of aortic rupture in LDS is significantly higher than in MFS [11]. EDS IV, resulting from *COL3A1* mutations causing type III collagen synthesis defects, leads to markedly reduced vascular wall tensile strength. Patients are prone to rupture of arteries, intestines, or the uterus, with aortic rupture being a primary cause of death [14].

Beyond these syndromic AAs, recent genomic studies have expanded the landscape of heritable thoracic aortic diseases [4]. Approximately 10–15% of familial thoracic aortic aneurysm (FTAA) patients harbor genetic variations, including genes like ACTA2, MYH11, and SMAD3, beyond *TGFBR1/2* [15,16,17]. These genes are involved in smooth muscle cell (SMC) contractile function, ECM synthesis, or TGF-β signaling. Their mutation leads to SMC dysfunction and vascular wall instability, ultimately triggering aneurysm formation. The genetic pattern of familial AAA is not fully elucidated, but individuals with a first-degree relative affected by AAA have a markedly increased risk, suggesting the potential synergistic effect of multiple susceptibility genes [6,18].

### 2.2 Extracellular Matrix Remodeling and Disruption of Vascular Wall Integrity

The ECM, comprising elastic fibers, collagen (primarily types I and III), and proteoglycans, is the main structural component of the aortic wall. The dynamic balance between its synthesis and degradation maintains vascular elasticity and tensile strength [3]. A core pathological feature of AA is abnormal ECM remodeling, characterized primarily by elastic fiber degradation, collagen proportion imbalance, and aberrant activation of the matrix metalloproteinase (MMP) family [3,5].

**Fig. 2.**
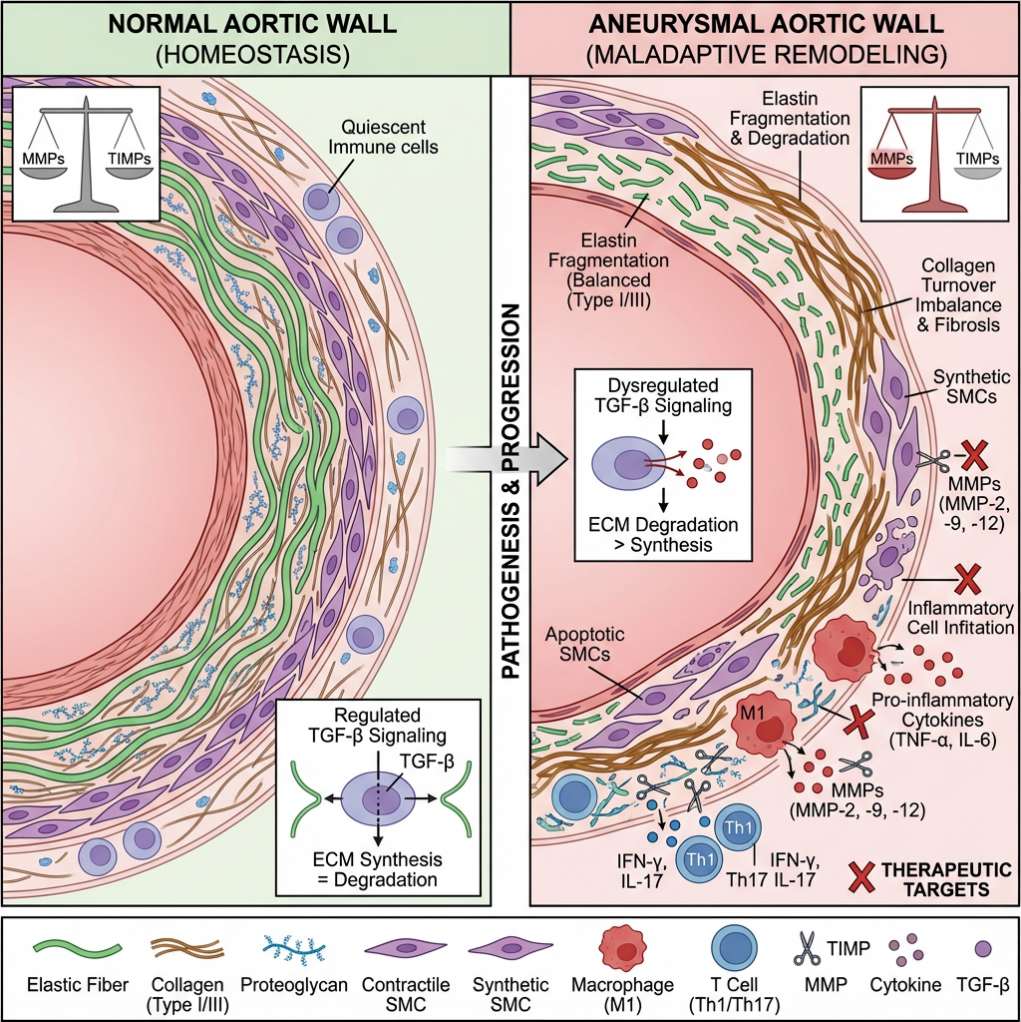
**Dysregulated extracellular matrix (ECM) remodeling in aortic aneurysm**. A comparison of the aortic media in a normal state versus an aneurysmal state. The normal architecture, with organized elastic fibers and collagen, is disrupted in the aneurysm wall. Key features include fragmented and diminished elastic fibers, an altered collagen I/III ratio, and an imbalance between matrix metalloproteinases (MMPs) and their inhibitors (TIMPs), leading to net ECM degradation. Concomitant smooth muscle cell (SMC) phenotypic switching and apoptosis further compromise the vascular wall’s structural integrity.

In AAA tissue, the number of elastic fibers is significantly reduced, and their structure is fragmented. Total collagen content increases, but the ratio is abnormal, with an increased ratio of type I to type III collagen, leading to reduced elasticity and increased brittleness of the vascular wall (Fig. 2). This process is primarily mediated by MMPs, zinc-dependent proteases capable of degrading ECM components. MMP-2, MMP-9, and MMP-12 are significantly upregulated in AA tissue [3]. Animal studies confirm that MMP activation directly leads to elastic fiber degradation and collagen hydrolysis, promoting aneurysm formation and expansion. Conversely, the expression of tissue inhibitors of metalloproteinases (TIMPs), natural inhibitors of MMPs, is significantly decreased in AA tissue, further exacerbating ECM degradation.

Emerging evidence suggests that iron dysregulation and oxidative stress may further exacerbate this process by promoting MMP activation and vascular cell death [19,20,21]. Furthermore, alterations in micromechanical properties of elastic lamellae have been implicated in early failure of the vascular wall [22].

### 2.3 Inflammation and Immune Cell Infiltration

The inflammatory response is another core component of AA pathogenesis. AAA tissue exhibits extensive infiltration of immune cells, including macrophages, T lymphocytes, B lymphocytes, and neutrophils. These cells participate in vascular wall injury and remodeling by releasing inflammatory cytokines and proteases [3].

Macrophages are the predominant inflammatory cells in AA tissue, and their infiltration degree correlates with aneurysm size and expansion rate. Macrophages can be classified into M1 and M2 types. M1 macrophages release pro-inflammatory cytokines like tumor necrosis factor-alpha (TNF-α) and interleukin-6 (IL-6), activating inflammatory responses and inducing MMP expression; M2 macrophages are primarily involved in tissue repair, but their function is dysregulated in AA, failing to effectively curb inflammation and ECM degradation. Notably, recent studies have highlighted the “gut-vascular axis”, suggesting that gut microbiota dysbiosis (e.g., changes in Faecalibacterium prausnitzii abundance) may modulate systemic inflammation and contribute to aneurysm development [23,24]. Disruption of the intestinal mucosal barrier and age-related changes in microbiota have also been linked to vascular inflammation [25,26].

T lymphocytes primarily play an immunomodulatory role in AA inflammation. Both CD4+ and CD8+ T cells are heavily infiltrated in AAA tissue. CD4+ T cells can differentiate into subsets like Th1, Th2, and Th17. Th1 cells release pro-inflammatory cytokines like interferon-gamma (IFN-γ), promoting macrophage activation; Th17 cells release IL-17, which can induce SMC apoptosis and MMP expression. Dysfunction of regulatory T cells (Tregs), which maintain immune balance by suppressing excessive inflammation, may also contribute to AA pathogenesis. A decrease in Treg number or function might lead to uncontrolled inflammation [3].

The network regulation of inflammatory cytokines plays a key role in AA progression. Serum levels of inflammatory markers like TNF-α, IL-6, and C-reactive protein (CRP) are significantly elevated in AAA patients and correlate with aneurysm size, expansion rate, and rupture risk [27].

### 2.4 The Central Role of the TGF-β Signaling Pathway

The transforming growth factor-beta (TGF-β) signaling pathway is a key regulator of vascular wall homeostasis and plays a dual role in AA pathogenesis. Its dysfunction is closely linked to both inherited and sporadic AAs [11].

TGF-β pathway activation involves TGF-β ligands binding to cell membrane receptors. In the normal vascular wall, the TGF-β pathway helps maintain ECM homeostasis by promoting collagen and elastic fiber synthesis; in AA, TGF-β pathway dysfunction manifests in two patterns: in genetic disorders like MFS and LDS, mutations in *TGFBR1/2* or SMAD3 lead to TGF-β pathway overactivation, inducing inflammatory responses and abnormal ECM remodeling [11]; in sporadic AAA, the TGF-β pathway may be suppressed, leading to inadequate ECM synthesis and impaired vascular wall repair capacity [6].

Animal studies confirm that inhibiting the overactive TGF-β pathway significantly delays aortic expansion in MFS mice; conversely, in AAA mouse models, moderate activation of the TGF-β pathway can promote ECM synthesis and slow aneurysm progression [6]. This dual role suggests that TGF-β pathway modulation requires precise intervention based on the AA subtype and pathological state, offering new avenues for targeted therapy.

### 2.5 Impact of Environmental and Comorbid Factors

Environmental risk factors and comorbid conditions are significant contributors to sporadic AA, primarily including smoking, hypertension, chronic obstructive pulmonary disease (COPD), diabetes, and obesity. Social determinants of health also play a role in the development of cardiovascular-kidney-metabolic syndromes associated with AA [12,28].

Smoking is the strongest modifiable risk factor for AA. Smokers have a 2–6 times higher risk of developing AAA compared to non-smokers, with a dose-response relationship [2]. Harmful substances in tobacco, such as nicotine and tar, directly damage endothelial cells, induce oxidative stress and inflammation, activate MMP expression, and promote ECM degradation [29]; simultaneously, smoking can inhibit the vascular wall’s repair capacity and reduce collagen synthesis, further weakening the wall structure. Studies confirm that smoking cessation significantly reduces the expansion rate and rupture risk of AAA, making it a key non-surgical management measure.

Hypertension is another major risk factor. Chronic hypertension increases shear stress on the vascular wall, damaging endothelial cells and SMCs, and activating inflammation and ECM remodeling. Hypertensive patients show more pronounced elastic fiber fragmentation in the aortic media, higher MMP expression levels, and faster aneurysm progression. Controlling blood pressure reduces mechanical stress on the vessel wall, decreases inflammatory cytokine release, and slows aneurysm expansion. Guidelines recommend a target blood pressure below 140/90 mmHg for AA patients, with a lower target of 130/80 mmHg for high-risk patients [30]. Obesity is another growing concern, contributing to cardiovascular aging and aneurysm risk through systemic inflammation [31].

COPD is significantly associated with an increased risk of AA. Approximately 7–11% of COPD patients have AAA, possibly due to the systemic effects of chronic inflammation. The systemic inflammatory state in COPD patients may lead to increased inflammatory cell infiltration in the vessel wall and MMP activation, promoting ECM degradation; additionally, the higher prevalence of smoking among COPD patients further increases AA risk [2]. Interestingly, diabetes is associated with a lower risk of developing AA, potentially due to vasculoprotective effects of insulin [32]; however, diabetic patients face a higher risk of postoperative complications, necessitating careful perioperative management.

## 3. Diagnosis and Screening of Aortic Aneurysm

Early diagnosis and accurate assessment of AA are crucial for reducing rupture risk and improving outcomes. The diagnostic process involves risk factor identification, clinical evaluation, and imaging, with well-implemented screening strategies significantly improving early detection rates [30].

### 3.1 Clinical Assessment and Risk Factor Identification

Clinical assessment for AA should first focus on the patient’s risk factor exposure history, including age, sex, smoking history, family history, hypertension, COPD, and diabetes [2,6]. Age is a significant risk factor, with AAA incidence markedly increasing in individuals over 65 years old. The prevalence is 3–4 times higher in men (4–8%) than in women (0.5–2%); a positive family history substantially increases risk; individuals with a first-degree relative affected by AAA have a screening detection rate as high as 30% [6].

Clinical symptoms of AA relate to the aneurysm’s location, size, and whether it has ruptured. Most AAA patients are asymptomatic in the early stages, often discovered incidentally during physical examination or imaging; larger aneurysms or those compressing surrounding tissues may cause vague abdominal pain, back pain, or abdominal distension. TAA patients may experience chest pain, chest tightness, or dyspnea. Compression of the airway, esophagus, or nerves can lead to cough, dysphagia, or hoarseness [8]. The classic triad for rupture is sudden, severe abdominal/chest pain, hypotension, and a pulsatile abdominal mass, indicating a medical emergency requiring immediate treatment.

Physical examination has limited diagnostic value for AA. Many AAAs are not palpable due to deep location or patient obesity; only about 30–40% of AAA patients present with a pulsatile mass on abdominal palpation. However, a physical exam may reveal signs of peripheral arterial disease (e.g., diminished lower limb pulses), which is associated with an increased risk of AA and warrants further screening. For suspected rupture, the physical exam should rapidly assess vital signs, abdominal tenderness, and pulsatile mass to determine severity [30]. In some cases, clinical features may overlap with other vascular conditions, requiring careful differential diagnosis [33].

### 3.2 Application and Comparison of Imaging Techniques

**Fig. 3.**
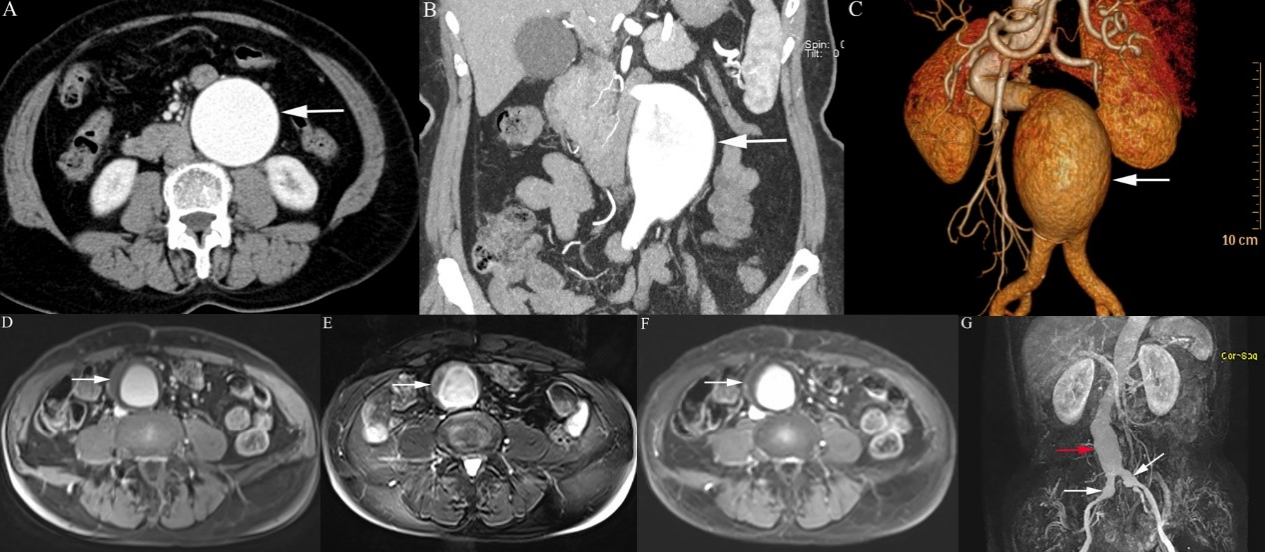
**Multimodality imaging assessment of abdominal aortic aneurysms**. (A) Axial CTA image, (B) coronal maximum intensity projection (MIP), and (C) volume-rendered (VR) reconstruction demonstrating an infrarenal AAA (white arrows) with clear visualization of the aneurysm neck and iliac arteries. (D) Axial T1-weighted image (T1WI), (E) T2-weighted image (T2WI), and (F) contrast-enhanced T1WI showing an infrarenal AAA (white arrows) with a filling defect consistent with intraluminal thrombus. (G) Coronal three-dimensional reconstruction image reveals the AAA (red arrow) and bilateral iliac artery aneurysms (white arrows). CTA, computed tomography angiography.

Imaging is the cornerstone of AA diagnosis and assessment. Common techniques include ultrasound, CTA, and MRA, each with advantages and specific scenarios for use (Fig. 3).

Ultrasound is the preferred imaging modality for screening and surveillance due to its non-invasive nature, convenience, low cost, and lack of ionizing radiation. It accurately measures aneurysm diameter, extent, and assesses the presence of thrombus [34]. Its sensitivity for detecting AAA ≥3 cm exceeds 95%, with near-perfect specificity. Limitations include interference from patient obesity or bowel gas, difficulty visualizing anatomic details like renal artery origins or iliac involvement, limited value for TAA, and inability to reliably diagnose rupture.

CTA is the gold standard for preoperative assessment, providing detailed visualization of aortic anatomy, aneurysm location, size, morphology, neck length and angle, and involvement of branch vessels, which is critical for surgical planning [35]. Its high spatial resolution allows for 3D reconstruction, accurately assessing wall calcification, thrombus, and surrounding tissue invasion; for suspected rupture, CTA can quickly confirm the diagnosis, showing the rupture site, extent of hemorrhage, and involvement of adjacent structures. Drawbacks include the use of iodinated contrast (risk of allergy), ionizing radiation, making it unsuitable for patients with renal impairment or pregnancy [30].

MRA is an important complementary technique, particularly for patients with renal insufficiency, contrast allergy, or when avoiding radiation is necessary [1]. MRA provides anatomic detail comparable to CTA, accurately measures aneurysm parameters, and can assess wall inflammation; contrast-enhanced MRA offers superior hemodynamic assessment, effectively identifying complications like endoleak. Recent advances include 4D flow MRI for functional assessment of hemodynamics [36]. Disadvantages include longer scan times, making it less suitable for unstable patients, and incompatibility with certain metallic implants [30].

Other techniques like digital subtraction angiography (DSA), once a primary diagnostic tool, are now primarily used for intraoperative imaging due to their invasive nature, high contrast load, and radiation dose [2]. Recently, techniques like cone-beam CT and contrast-enhanced ultrasound are increasingly used. Contrast-enhanced ultrasound improves the detection of small aneurysms and endoleaks, while cone-beam CT provides rapid intraoperative 3D anatomic information.

### 3.3 Screening Strategies and Guideline Recommendations

Screening for AA is key to early detection and reducing rupture risk. Population-based screening strategies can significantly reduce AAA-related mortality. Current national and international guidelines recommend screening primarily based on risk stratification, focusing on high-risk populations [30].

The U.S. Preventive Services Task Force (USPSTF) recommends one-time ultrasound screening for men aged 65–75 with a history of smoking; for men who never smoked or for women, routine screening is not recommended, but selective screening may be considered for women with a family history or smoking history. The Society for Vascular Surgery (SVS) guidelines offer broader recommendations, suggesting one-time ultrasound screening for men aged 65 and older, men aged 55 and older with a family history of AAA, and women aged 65 and older with a family history or smoking history [30]. European guidelines recommend universal screening for men aged 65–74, reserving screening for women only with a family history or smoking history.

**Fig. 4.**
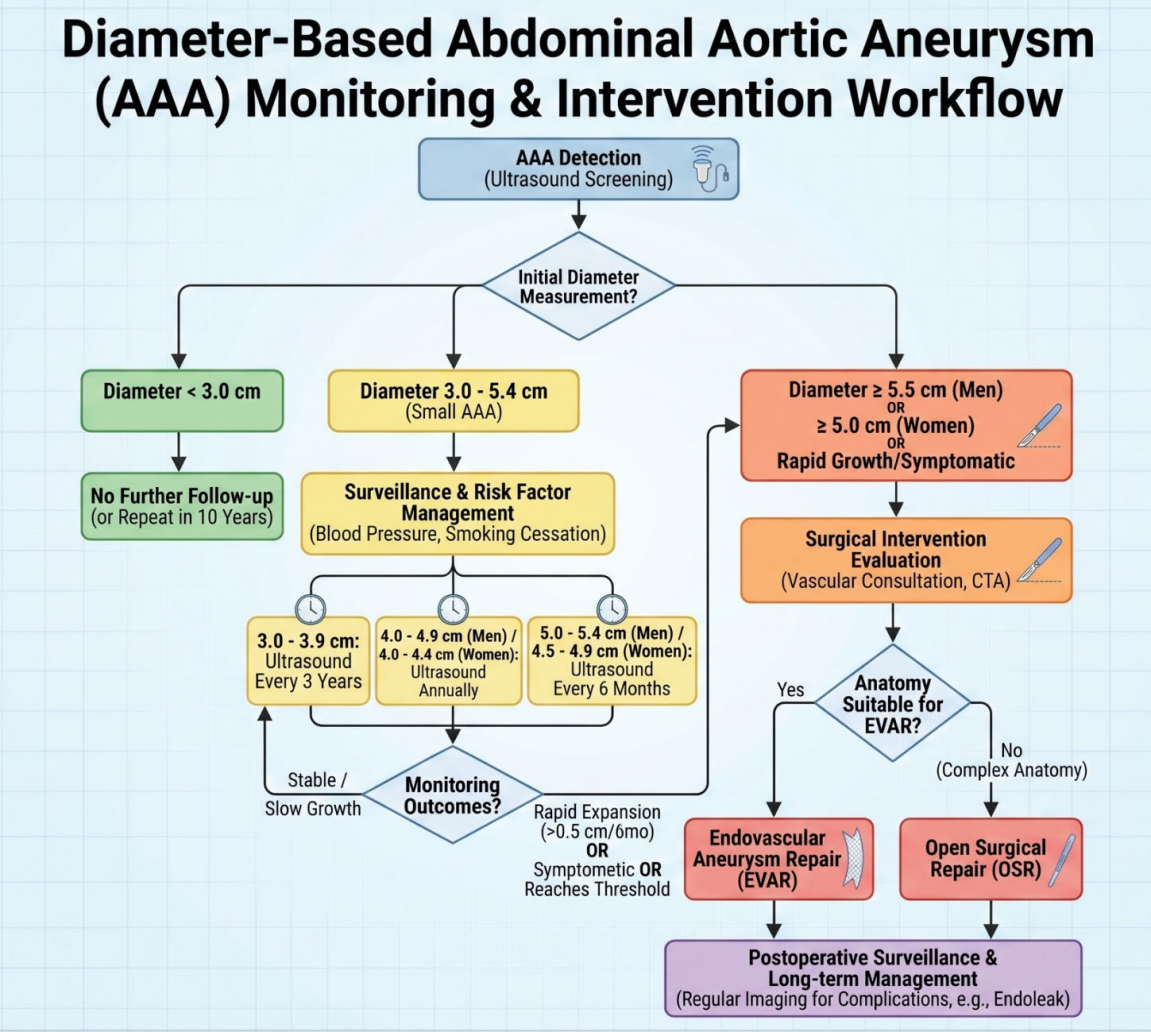
**Evidence-based management algorithm for abdominal aortic aneurysm (AAA) based on maximum diameter**. This flowchart outlines the recommended surveillance intervals and intervention thresholds for AAA identified through screening. The pathway is initiated by the detection of an AAA and is primarily driven by its maximum diameter, with accelerated expansion (>0.5 cm/6 months) or the onset of symptoms prompting re-evaluation for intervention regardless of size. Note the lower diameter threshold for surgical consideration in female patients.

Management of screening results is stratified by aortic diameter, guiding surveillance intervals and intervention decisions (Fig. 4). Specifically, individuals with an aortic diameter <3 cm require no further follow-up or a repeat scan in 10 years; patients with AAA diameters of 3.0–3.9 cm should undergo ultrasound every 3 years; 4.0–4.9 cm, annually; 5.0–5.4 cm, every 6 months; men with diameters ≥5.5 cm or women with diameters ≥5.0 cm should be evaluated promptly for surgical intervention [30]. Additionally, patients with an expansion rate >0.5 cm/6 months require intensified monitoring or consideration for intervention, regardless of absolute diameter.

Screening implementation should consider cost-effectiveness and population characteristics. In regions with high smoking rates, screening in male populations is more cost-effective [37]; given the lower incidence in women, screening only high-risk women is more reasonable. Recently, the use of electronic health records and “best practice alert” systems has significantly improved screening coverage and reduced missed diagnoses. Recent data suggests that social determinants of health should also be considered when designing screening programs to ensure equitable access [28].

## 4. Clinical Management of Aortic Aneurysm

The clinical management of AA is a comprehensive process aimed at preventing rupture and improving patient outcomes. It encompasses surgical intervention, pharmacological therapy, and complication management, with treatment plans individualized based on aneurysm characteristics, comorbidities, and age [30].

### 4.1 Surgical Treatment: Evolution of Open and Endovascular Repair

**Fig. 5.**
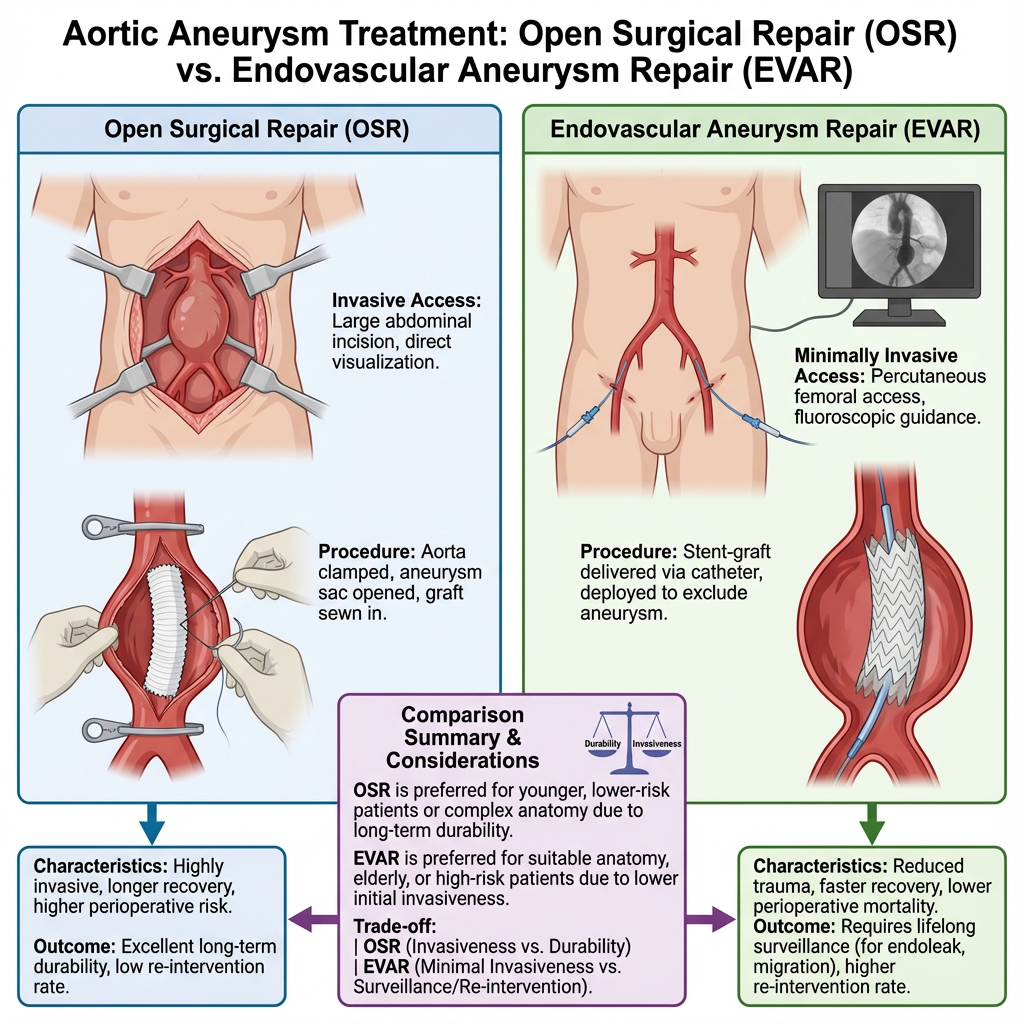
**Schematic comparison of open surgical repair (OSR) and endovascular aneurysm repair (EVAR)**. **Left.** OSR for an abdominal aortic aneurysm (AAA) involves a laparotomy, direct dissection and opening of the aneurysm, and the implantation of a prosthetic graft, which is sutured in place above and below the aneurysmal segment. **Right.** EVAR is a minimally invasive procedure where a stent-graft is delivered via the femoral artery under fluoroscopic guidance. The stent-graft is deployed within the aorta, effectively excluding the aneurysm from systemic pressure and inducing thrombus formation within the sac. Key anatomical landmarks and procedural steps are illustrated.

Surgical intervention is the cornerstone of AA treatment, primarily consisting of open surgical repair (OSR) and endovascular aneurysm repair (EVAR), with TEVAR being the application of EVAR principles to the thoracic aorta (Fig. 5). The core principle of both approaches is to eliminate the risk of rupture by placing a graft or prosthetic conduit to exclude the aneurysmal segment.

OSR is the traditional approach. For AAA, OSR involves accessing the aorta via an abdominal incision (transperitoneal or retroperitoneal), exposing the aneurysm, resecting the aneurysmal segment, and implanting a prosthetic graft to restore blood flow. For TAA, OSR requires a thoracic or thoracoabdominal incision to expose the thoracic aorta for graft replacement. The advantages of OSR include direct visualization, ability to handle complex anatomies, excellent long-term durability, and low re-intervention rates [13]. However, OSR is highly invasive, carries a higher perioperative mortality rate (3–6%), higher risk of postoperative complications (e.g., pulmonary infection, renal insufficiency, bowel ischemia), and longer hospital stays compared to EVAR.

EVAR is a minimally invasive technique involving percutaneous access via the femoral or brachial arteries. A stent-graft is delivered through the vasculature to the aneurysm site, deployed to exclude the aneurysm from systemic pressure. EVAR offers advantages of reduced trauma, lower perioperative mortality (1–2%), and faster recovery, making it the preferred treatment for AAA patients with suitable anatomy [13,38]. Similarly, TEVAR has significantly improved outcomes for TAA patients, especially high-risk cases or those with complex anatomy [8]. However, EVAR is limited by anatomic constraints, requiring adequate proximal neck length (≥10–15 mm), favorable neck angle (<60°), and absence of severe calcification or thrombus. Long-term monitoring is necessary for complications like endoleak, stent migration, and graft infection.

Advances in complex EVAR techniques have significantly expanded the indications for endovascular treatment. These include chimney EVAR (ch-EVAR), fenestrated EVAR (f-EVAR), and branched stent-graft techniques, which can treat juxtarenal, pararenal, or thoracoabdominal aneurysms, addressing anatomic challenges not suitable for standard EVAR [39]. ch-EVAR involves placing parallel stents alongside the main graft to preserve flow to visceral arteries; f-EVAR uses custom grafts with fenestrations (holes) aligned precisely with visceral artery origins. Studies confirm high technical success rates (91–100%) for complex EVAR, with 30-day mortality below 3.4%, but re-intervention rates are relatively higher (around 15%), necessitating diligent long-term follow-up [39].

### 4.2 Controversies in Surgical Indications and Timing

The selection of surgical candidates and optimal timing are central controversies in AA management. Current guideline recommendations are primarily based on aneurysm diameter, expansion rate, symptoms, and individual patient factors, but several issues remain debated [30].

Aneurysm diameter is the primary criterion for determining the timing of elective surgery. Guidelines recommend elective repair for men with AAA diameters ≥5.5 cm and women with diameters ≥5.0 cm [30]. This is based on studies showing the annual rupture risk for AAA ≥5.5 cm is high (9.4–32.5%), and the benefit of surgery outweighs the risk. The threshold is lower for women because female AAA has a higher rupture risk for a given size, and ruptures often occur at smaller diameters. For men with diameters of 5.0–5.4 cm and women with 4.5–4.9 cm, timing should be individualized, considering expansion rate, age, and comorbidities.

Aneurysm expansion rate is another important factor. Guidelines suggest intervention for expansion >0.5 cm/6 months [30]. Rapid expansion significantly increases rupture risk, warranting intervention even if the diameter threshold is not met. Symptomatic AA patients (e.g., with abdominal or back pain) require prompt evaluation for surgery regardless of size, as symptoms often indicate impending rupture.

The need for early surgery in asymptomatic patients with small AAA (<5.5 cm) remains controversial. The UK Small Aneurysm Trial and Aneurysm Detection and Management (ADAM) Trial found that early surgery did not improve long-term survival for small AAA patients and increased perioperative mortality, thus recommending surveillance over early intervention [40]. However, recent studies suggest that early EVAR might reduce long-term rupture risk and improve outcomes for younger, low-risk patients [40]. The timing of intervention for small AAAs in patients undergoing other major procedures (e.g., chemotherapy, organ transplant) requires shared decision-making, balancing surgical risk against the risk of disease progression.

### 4.3 Current Status and Potential Targets of Pharmacological Therapy

Pharmacological therapy is an important component of comprehensive AA management, primarily aiming to control risk factors, slow aneurysm progression, and reduce postoperative complication risks. However, currently, no specific drug is approved to arrest aneurysm growth [3,5].

Current recommended pharmacological management focuses on controlling blood pressure, lipids, and smoking cessation. Antihypertensive therapy reduces mechanical stress on the vessel wall, inflammation, and ECM degradation. Guidelines recommend using angiotensin-converting enzyme (ACE) inhibitors, angiotensin receptor blockers (ARBs), beta-blockers, or diuretics to achieve a target blood pressure <140/90 mmHg [30]. Statins, with their anti-inflammatory, antioxidant, and MMP-inhibiting properties, reduce cardiovascular complication risks in AA patients. While some studies suggest statins may slow AAA expansion, guidelines do not recommend their use solely for this purpose [30]. Smoking cessation is a critical non-surgical measure, significantly reducing expansion rate and rupture risk, and should be counseled at every follow-up.

Research into potential targeted therapies is active, focusing on inflammation, ECM remodeling, and the TGF-β pathway. MMP inhibitors (e.g., doxycycline) can inhibit ECM degradation; animal studies showed delayed AAA progression, but clinical trials have not confirmed significant efficacy [41]. TGF-β pathway modulators show promise, particularly for genetic AAs; clinical studies in MFS patients confirmed that losartan (an ARB) can inhibit the overactive TGF-β pathway and delay aortic expansion [11]. Anti-inflammatory drugs like IL-1β inhibitors or TNF-α inhibitors can suppress inflammation; animal studies are promising, but clinical research is ongoing.

Other agents, like antibiotics (e.g., roxithromycin) targeting Chlamydia pneumoniae-associated inflammation, failed to show benefit in clinical trials. Beta-blockers (e.g., propranolol) were tested but multiple randomized controlled trials (RCTs) found no significant effect on AAA progression and poor tolerability. Recent experimental and observational evidence suggests that anti-diabetic drugs, specifically metformin and GLP-1 receptor agonists, may be associated with reduced AAA growth, potentially offering a new therapeutic avenue [32,42,43]. Lifestyle interventions, such as physical activity, also show potential benefits in modulating cardiometabolic risk factors [44]. Exercise training has been suggested to be safe and effective for patients with small AAAs [45]. Future drug development should focus on precision targeting based on patient genotype and pathological phenotype for individualized therapy.

### 4.4 Diagnosis and Management of Complications

Complications of AA, including intraoperative and postoperative events, significantly impact prognosis. Timely diagnosis and effective management are crucial for improving outcomes.

Intraoperative complications include hemorrhage, vascular injury, and visceral ischemia. Major bleeding, the most serious complication during OSR, often due to fragile vessels or anastomotic failure, requires prompt hemostasis, blood product transfusion, and possibly cell salvage techniques. Vascular injuries like dissection or perforation are more common during EVAR and require management with balloon inflation or additional stent placement [46]. Visceral ischemia (e.g., bowel, renal) can result from vessel clamping or embolism; preoperative assessment of visceral anatomy, minimizing clamp time, and postoperative monitoring can reduce its incidence.

**Fig. 6.**
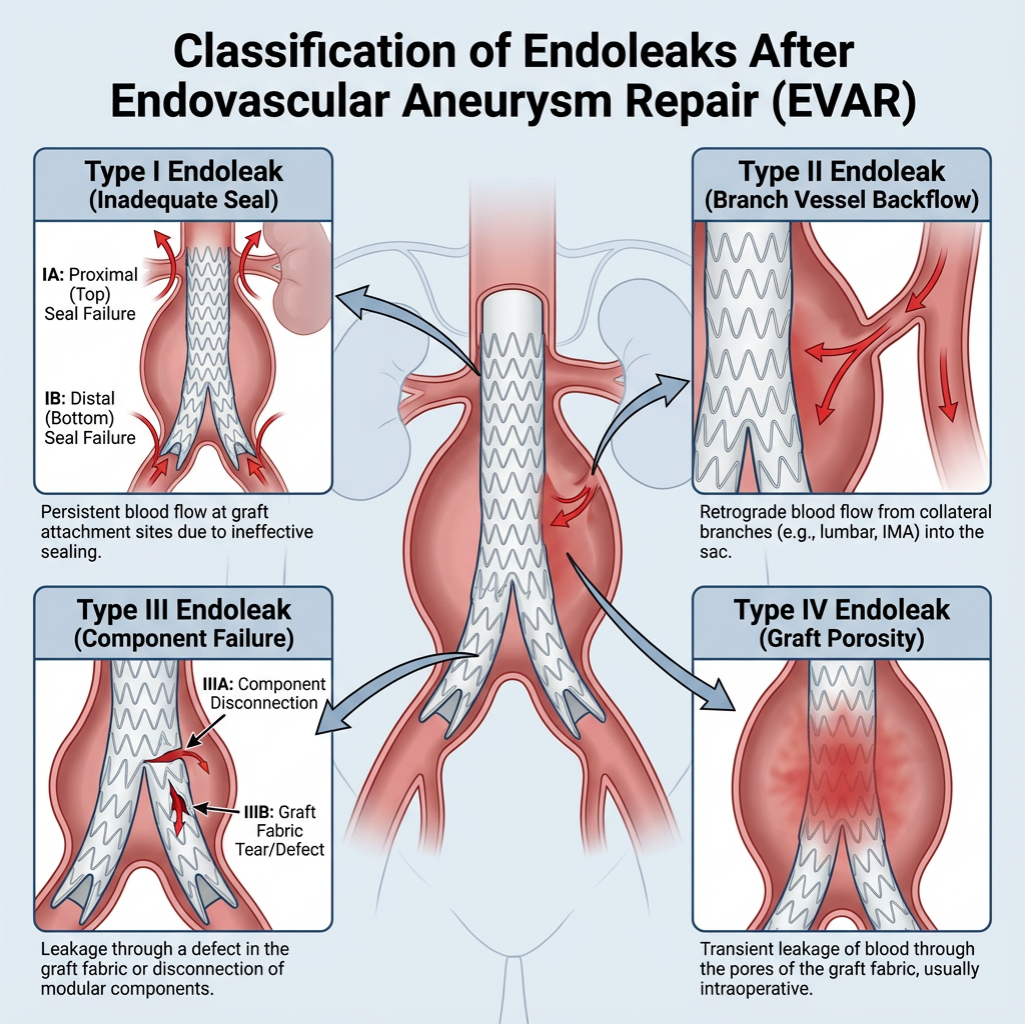
**Classification of endoleaks after endovascular aneurysm repair**. Schematic illustrations of the four primary types of endoleak, characterized by the source of persistent blood flow into the excluded aneurysm sac. **Type I:** Inadequate seal at the proximal (Ia) or distal (Ib) attachment zones. **Type II:** Retrograde perfusion from patent branch arteries (e.g., inferior mesenteric artery or lumbar arteries). **Type III:** Leakage due to component separation or fabric tear. **Type IV:** Leakage through the graft fabric, often seen during implantation and usually transient. Types I and III are considered high-risk and typically require intervention, whereas Type II may be managed conservatively with surveillance. IMA, inferior mesenteric artery.

Postoperative complications include endoleak, stent migration, graft infection, bowel ischemia, and renal dysfunction. Endoleak, the most common complication after EVAR, is defined as persistent blood flow outside the stent-graft lumen but within the aneurysm sac (Fig. 6). Types include I (inadequate seal at proximal or distal attachment sites), II (retrograde flow from branch vessels, such as the inferior mesenteric or lumbar arteries), III (disconnection or tear of the stent-graft fabric), and IV (blood flow through the graft fabric pores, typically self-limiting) [46]. Type I and III endoleaks usually require prompt treatment (e.g., balloon angioplasty, stent extension, embolization). Type II endoleaks can often be monitored if the aneurysm is stable but may need intervention if associated with sac expansion [46]. Stent migration, often due to unfavorable anatomy or inappropriate device selection, may require stent extension or conversion to open repair.

Graft infection is a serious complication (~0.3% incidence), presenting with fever, chills, wound issues, or pseudoaneurysm formation. It requires prompt antibiotic therapy and potentially surgical removal of the infected graft and vascular reconstruction [47]. Bowel ischemia is a serious complication after OSR (1–3% incidence), presenting with abdominal pain, diarrhea, or bloody stools. Diagnosis is confirmed by endoscopy; mild cases may be managed conservatively, while severe cases require bowel resection. Renal dysfunction can result from intraoperative ischemia or contrast-induced nephropathy. Preoperative hydration, renal protection during surgery, and postoperative monitoring can reduce its risk.

## 5. Controversies and Unresolved Challenges

Despite significant progress, several controversies and unresolved issues persist in AA management, directly impacting clinical decision-making and patient outcomes [30].

### 5.1 Optimizing Screening Strategies

Current screening strategies primarily rely on traditional risk factors like age, sex, smoking history, and family history. However, some high-risk individuals might be missed. For example, should younger individuals (<65) with a family history of AA or individuals without smoking history but with multiple other risk factors (e.g., hypertension, COPD) be included in screening programs? Furthermore, the optimal screening interval and the necessity of repeat screening are debated. While a 10-year rescreening interval might suffice for those with a diameter <3 cm, shorter intervals might be needed for those with a strong family history or prolonged smoking history.

### 5.2 Controversies in Surgical Timing and Modality Selection

For male AAA patients with diameters between 5.0–5.5 cm, the choice of surgical timing requires balancing operative risk against rupture risk. Younger, lower-risk patients might benefit from earlier intervention, while older, higher-risk patients might be better served by intensive surveillance [40]. The choice of treatment for complex anatomy aneurysms (e.g., juxtarenal, thoracoabdominal) is also contentious. OSR offers more durable long-term results but is highly invasive. Complex EVAR is minimally invasive but carries a higher re-intervention rate, necessitating individualized decisions based on specific patient circumstances [39].

### 5.3 Efficacy of Pharmacological Therapy

There are currently no specific drugs that can significantly delay AA progression; the main benefits of statins, ACE inhibitors, and beta-blockers lie in controlling cardiovascular complications rather than directly inhibiting aneurysm expansion [30]. Clinical studies on potential targeted drugs such as MMP inhibitors and anti-inflammatory drugs have not yielded satisfactory results, possibly due to drug specificity, dose selection, treatment timing, and other factors [41]. Future drug therapy requires more precise target identification and individualized regimen development.

### 5.4 Postoperative Monitoring and Complication Management Controversies

The frequency and methodology of postoperative surveillance following EVAR remain subjects of debate. Guidelines typically recommend computed tomography angiography (CTA) scans at 1 month, 6 months, and 12 months post-procedure, followed by annual imaging [30]. However, some studies suggest that if the 1-month scan shows no abnormalities, the 6-month CTA might be omitted in favor of ultrasound surveillance, potentially reducing radiation exposure and contrast-induced nephropathy risk, especially in patients with renal impairment. The management of endoleaks is particularly contentious. While type I and III endoleaks generally require intervention due to the persistent risk of rupture, the management of type II endoleaks is less clear-cut. Some asymptomatic type II endoleaks can be monitored safely over the long term if the aneurysm sac is stable or shrinking. However, intervention is warranted if associated with sac expansion (>5 mm), but the optimal timing and method of intervention (e.g., transarterial embolization, laparoscopic ligation of collateral vessels) lack standardized consensus [46]. The choice of monitoring technology also involves trade-offs. While CTA is the traditional standard for detecting endoleaks and measuring sac diameter, concerns about cumulative radiation dose and nephrotoxic contrast have prompted the exploration of alternatives. Duplex ultrasonography is a valuable, non-invasive tool for follow-up, but its accuracy can be operator-dependent and limited by patient body habitus or bowel gas [34]. Contrast-enhanced ultrasound (CEUS) has been shown to improve the sensitivity for detecting endoleaks compared to standard duplex and may be comparable to CTA for this purpose, offering a radiation-free alternative. Furthermore, magnetic resonance imaging (MRI), particularly with specific contrast protocols, may offer superior sensitivity for detecting certain types of endoleaks compared to CTA, but its higher cost, longer scan time, and contraindications in patients with certain metallic implants limit its widespread use for routine surveillance. The evolving landscape of imaging technologies necessitates ongoing re-evaluation of surveillance protocols to optimize patient safety and diagnostic accuracy.

## 6. Future Perspectives: Precision Medicine and Technological Innovation

The diagnosis and treatment of AA are poised to advance towards greater precision, minimal invasiveness, and personalization. The integration of basic research and clinical technology will provide new insights to address current controversies and challenges [3,6].

### 6.1 Application of Precision Medicine in AA Management

Advances in genetic sequencing technologies will enable more precise identification of causative genes in hereditary AA, facilitating early screening and risk stratification through genetic testing [6]. For instance, genetic testing in patients with familial AA can identify the specific pathogenic mutation, providing a basis for genetic counseling and screening recommendations for relatives. Studies on outcomes of genetic counseling have shown its importance in patient management [48]. Genetic testing in sporadic AA patients may identify underlying genetic susceptibility factors, enabling personalized risk assessment [3,6]. Furthermore, selecting targeted therapies based on a patient’s genetic profile and pathological phenotype will significantly improve the efficacy of pharmacological treatments. Examples include using losartan for patients with overactive TGF-β signaling pathways or specific MMP inhibitors for patients with high MMP expression [11]. Epigenetic regulation is also emerging as a key factor in AA development, offering new potential biomarkers [49].

### 6.2 Development of Novel Therapeutic Targets

Deeper basic research will uncover more critical targets in AA pathogenesis, such as key cytokines in inflammation (e.g., IL-17, TNF-α), key enzymes in ECM remodeling (e.g., MMP-9, TIMP-1), and regulators of vascular wall cell phenotypic switching [3]. Novel drugs targeting these pathways (e.g., monoclonal antibodies, small molecule inhibitors) will enter clinical trials, potentially offering new options for AA pharmacotherapy. Identifying biomarkers for vascular aging in aortic tissues may provide early targets for intervention [50]. Additionally, innovative approaches like cell therapy (e.g., mesenchymal stem cell transplantation) and gene therapy (e.g., silencing MMP genes, overexpressing TIMP-1) are under exploration. Animal studies have confirmed their potential to delay aneurysm progression, and they may eventually find clinical application.

### 6.3 Evolution of Imaging Technologies

Advancements in imaging will further enhance the precision of AA diagnosis and assessment. For example, three-dimensional ultrasound and elastography techniques can assess vessel wall stiffness and elasticity, potentially predicting aneurysm expansion rate and rupture risk by analyzing local variations in material properties [51]. Positron emission tomography-computed tomography (PET-CT) can evaluate the degree of inflammation in the vessel wall, providing valuable information for disease assessment and monitoring treatment response. Functional assessment of aneurysms is becoming increasingly important for risk stratification [36]. The application of artificial intelligence (AI) can enable automated measurement of aneurysm parameters, intelligent analysis of anatomical structures, and computer-assisted surgical planning, thereby improving diagnostic and therapeutic efficiency and accuracy.

### 6.4 Optimization of Minimally Invasive Techniques

Continuous refinement of EVAR technology will expand the indications for endovascular therapy and improve surgical outcomes and safety. For example, the development of softer, more conformable stent-grafts that better adapt to vascular anatomy can reduce the incidence of stent migration and endoleak [39]. Rapid manufacturing technologies for customized fenestrated and branched stents can shorten waiting times for surgery and improve the treatment of complex aneurysms. Moreover, the adoption of robotic-assisted surgery and percutaneous EVAR techniques will further reduce surgical trauma and improve patient recovery.

## 7. Conclusion

Aortic aneurysm is a complex degenerative vascular disease whose pathogenesis involves multiple levels, including genetic factors, ECM remodeling, inflammatory responses, and signaling pathway dysregulation. Advances in diagnostic techniques have enabled early identification and precise assessment of the disease. The comprehensive application of surgical intervention and pharmacological treatment has significantly reduced patient mortality. However, the management of AA still faces numerous controversies and challenges, including the optimization of screening strategies, personalized selection of surgical timing and approach, the development of effective drugs, and the long-term management of postoperative complications.

In thefuture, with the continued development of precision medicine, novel targeted therapies, imaging technologies, and minimally invasive techniques, the management of AA will transition from a “one-size-fits-all” to a personalized approach. Genetic testing will enable early risk stratification, novel pharmacological interventions may slow disease progression, precise minimally invasive surgery will reduce treatment risks, and AI technologies will optimize clinical workflows, ultimately improving long-term patient outcomes. The close integration of basic research and clinical practice is expected to bring new breakthroughs in the diagnosis and treatment of AA, potentially enabling effective prevention, early diagnosis, and precise therapy.

## Abbreviations

AA, Aortic Aneurysm; AAA, Abdominal Aortic Aneurysm; ACEI, Angiotensin-Converting Enzyme Inhibitor; ARB, Angiotensin II Receptor Blocker; ch-EVAR, Chimney Endovascular Aneurysm Repair; COPD, Chronic Obstructive Pulmonary Disease; CRP, C-Reactive Protein; CTA, Computed Tomography Angiography; DSA, Digital Subtraction Angiography; ECM, Extracellular Matrix; EDS IV, Ehlers-Danlos Syndrome Type IV; EVAR, Endovascular Aneurysm Repair; f-EVAR, Fenestrated Endovascular Aneurysm Repair; FTAA, Familial Thoracic Aortic Aneurysm; IFN-γ, Interferon-Gamma; IL-1β, Interleukin-1 Beta; IL-6, Interleukin-6; LDS, Loeys-Dietz Syndrome; MFS, Marfan Syndrome; MMP, Matrix Metalloproteinase; MRA, Magnetic Resonance Angiography; MRI, Magnetic Resonance Imaging; OSR, Open Surgical Repair; PET-CT, Positron Emission Tomography-Computed Tomography; SMC, Smooth Muscle Cell; TAA, Thoracic Aortic Aneurysm; TEVAR, Thoracic Endovascular Aneurysm Repair; TGF-β, Transforming Growth Factor-Beta; TIMP, Tissue Inhibitor of Metalloproteinase; TNF-α, Tumor Necrosis Factor-Alpha; USPSTF, U.S. Preventive Services Task Force.
